# Recombinant Hepatitis E virus like particles can function as RNA nanocarriers

**DOI:** 10.1186/s12951-015-0101-9

**Published:** 2015-06-24

**Authors:** Subrat Kumar Panda, Neeraj Kapur, Daizy Paliwal, Hemlata Durgapal

**Affiliations:** Department of Pathology, All India Institute of Medical Sciences, Ansari Nagar, New Delhi, 110029 India

**Keywords:** Hepatitis E virus, Virus like particles, RNA-VLP complex, Chimeric RNA, HBsAg

## Abstract

**Background:**

Assembled virus-like particles (VLPs) without genetic material, with structure similar to infectious virions, have been successfully used as vaccines. We earlier described in vitro assembly, characterisation and tissue specific receptor dependent Clathrin mediated entry of empty HEV VLPs, produced from *Escherichia coli* expressed HEV capsid protein (pORF2). Similar VLP’s have been described as a potential candidate vaccine (Hecolin) against HEV.

**Findings:**

We have attempted to use such recombinant assembled Hepatitis E virus (HEV) VLPs as a carrier for heterologous RNA with protein coding sequence fused in-frame with HEV 5′ region (containing cap and encapsidation signal) and investigated, if the relevant protein could be expressed and elicit an immune response in vivo. In vitro transcribed red fluorescent protein (RFP)/Hepatitis B virus surface antigen (HBsAg) RNA, fused to 5′-HEV sequence with cap and encapsidation signal (1–249 nt), was packaged into the recombinant HEV-VLPs and incubated with five different cell lines (Huh7, A549, Vero, HeLa and SiHa). The pORF2-VLPs could specifically transfer exogenous coding RNA into Huh7 and A549 cells. In vivo, *Balb*/c mice were immunized (intramuscular injections) with 100 µg pORF2-VLP encapsidated with 5′-methyl-G-HEV (*1*–*249* *nt*)-*HBsAg* RNA, blood samples were collected and screened by ELISA for anti-pORF2 and anti-HBsAg antibodies. Humoral immune response could be elicited in *Balb*/*c* mice against both HEV capsid protein and cargo RNA encoded HBsAg protein.

**Conclusions:**

These findings suggest that other than being a possible vaccine, HEV pORF2-VLPs can be used as a promising non-replicative tissue specific gene delivery system.

## Findings

VLPs inherit most of the properties of parent virus, like capability of self-assembly into organised structure, specific interaction with nucleic acid/protein, and cell-specific entry. These replication-deficient, non-infectious, nano-structured particles can be useful if they can effectively deliver therapeutically useful nucleic acid, drug, targeting peptide or a conjugated imaging molecule.

We had described generation of HEV VLP’s from *Escherichia coli* produced capsid protein [1]. Transmission electron microscopy (TEM) and nanoparticle tracking analysis (a rapid and direct NTA technique for real-time visualization of nanoparticles in liquid) showed HEV VLP’s as uniform particles of ~30–35 nm in size, consistent with the size of infectious HEV virions. The specificity of HEV-VLP binding and entry into the liver cells was demonstrated using reporter linked fluorescent VLP’s [1]. Similar bacterially generated VLPs (HEV 239) have been licensed as a potential candidate vaccine (Hecolin) against HEV in China [2–4].

Here, we investigate whether (1) empty VLPs of HEV could encapsidate heterologous RNA fused with encapsidation signal and deliver the exogenous RNA in a cell specific manner as a nanocarrier? (2) Can the foreign gene be translated from delivered chimeric RNA? and (3) If injected to animals, can the RNA-VLP complex induce immunity to both the carrier HEV capsid protein and the protein expressed from delivered RNA? To study the above possibilities, we generated a chimeric RNA where reporter/antigen producing gene/coding sequence (RFP/HBsAg) is fused in-frame with the HEV 5′ RNA region containing cap and encapsidation signal.

### Encapsidation of HEV-VLPs with in vitro transcribed RNA

**Figure 1:**
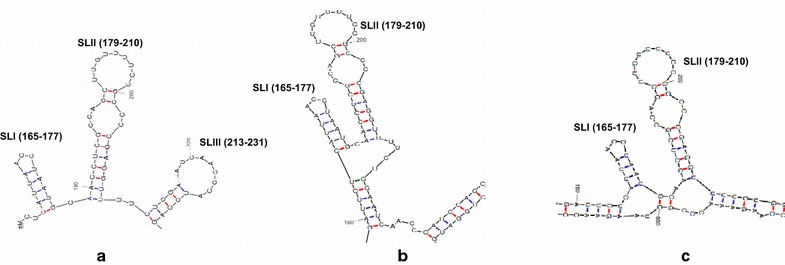
RNA secondary structure prediction using mfold program. Secondary structure prediction of **a** HEV (1–249 nt) RNA, **b** HEV (1–249 nt)-HBsAg RNA and **c** HEV (1–249 nt)-RFP RNA, using mfold program, based on free energy calculations.

Based on RNA secondary structure prediction software (mfold), it was found that HEV 5′-end [which contains 5′ non-coding region (NCR) of HEV (1–28 nt) and initial coding region of ORF1 (29–249 nt)] bears three stem-loop structures viz. SLI (165–177 nt), SLII (179–210 nt), and SLIII (213–231 nt) (Figure 1). These stem-loop structures are possibly responsible for interaction with HEV capsid protein. SLI and SLII are particularly important as similar structures (165–206 nt) are known to be conserved among most of the alphaviruses such as Sindbis, semliki Forest and Highlands J virus [5]. SLIII on the other hand, is not absolutely essential but may function to enhance the interaction of RNA with HEV capsid protein. We observed that the arrangement of HEV stem-loop structures SLI (165–177 nt) and SLII (179–210 nt), remained conserved even after in-frame fusion with foreign RNA (*RFP/HBsAg*), indicating their formation and stability in chimeric RNA (Figure 1).

**Figure 2:**
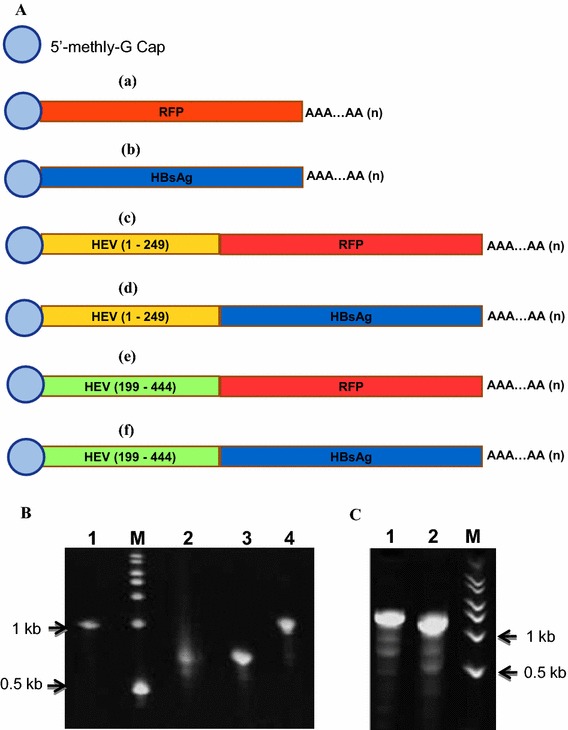
**A** Schematic representation of in vitro transcribed RNA constructs. *a* 5′-methyl-G-*RFP*, *b* 5′-methyl-G-*HBsAg*, *c* 5′-methyl-G-HEV-(*1*–*249* *nt*)-*RFP*, *d* 5′-methyl-G-HEV-(*1*–*249* *nt*)-*HBsAg*, *e* 5′-methyl-G-HEV-(*199*–*444* *nt*)-*RFP*, *f* 5′-methyl-G-HEV-(*199*–*444* *nt*)-*HBsAg*. **B** Urea gel representing integrity of various in vitro transcribed RNAs (with 5′ cap and 3′ poly-A tail). *Lane 1* 5′-methyl-G-HEV-(*1*–*249* *nt*)-*RFP* (947 bp); *lane 2* 5′-methyl-G-*RFP* (698 bp); *lane 3* 5′-methyl-G-*HBsAg*, (677 bp); *lane 4* 5′-methyl-G-HEV-(*1*–*249* *nt*)-*HBsAg* (926 bp); *lane M* RNA Millenium marker (Ambion). The size of all above transcribed RNAs in gel, seems greater by 30–50 nt, due to 3′ poly-A tail added to them. **C** Urea gel representing integrity of various in vitro transcribed RNAs (with 5′ cap and 3′ poly-A tail). *Lane 1* 5′-methyl-G-HEV-(*199*–*444* *nt*)-*RFP* (943 bp); *lane 2* 5′-methyl-G-HEV-(*199*–*444* *nt*)-*HBsAg*, (922 bp); *lane M* RNA Millenium marker (Ambion). The size of all above transcribed RNAs in gel, seems greater by 30–50 nt, due to 3′ poly-A tail added to them.

The capped RNA transcripts with poly(A) tail [*5′*-*methyl*-*G*-*RFP*, *5′*-*methyl*-*G*-*HBsAg*, *5′*-*methyl*-*G*-*HEV*(*1*–*249* *nt*)-*RFP*, *5′*-*methyl*-*G*-*HEV*(*199*–*444* *nt*)-*RFP*, *5′*-*methyl*-*G*-*HEV*(*1*–*249* *nt*)-*HBsAg* and *5′*-*methyl*-*G*-*HEV*(*199*–*444* *nt*)-*HBsAg*] (Figure 2A) were produced by T7 RNA polymerase (from their respective pGEMT easy-DNA constructs, Figure 2A) using mMessage mMachine T7 Ultra Transcription kit (Ambion) according to the manufacturer’s instruction and the integrity of synthesized RNA was checked on 5% urea-acrylamide gel (Figure 2B, C). The HEV, pORF2 and N-terminal (Δ1-112aa) deleted pORF2 VLPs were synthesized as described earlier [1], and encapsidated separately with different heterologous RNA molecules using direct interaction method [6]. After the packaging of RNA, HEV-VLP complexes were treated with RNase A to degrade the unpackaged RNA.

### Internalization of HEV-RNA-VLP complex into the cultured cells

To ascertain the feasibility of synthetic VLPs as a vehicle for nucleic acid delivery, we checked the expression of protein encoded from packaged foreign RNA (RFP/HBsAg) in five different cell lines i.e.; Huh7, A549, Vero, HeLa and SiHa. Freshly harvested cells (5 × 10^4^ cells) were plated, allowed to adhere and incubated separately with 250 nM (saturation binding concentration based on our earlier work [1]) of various RNA-VLP complexes. At various time points, post incubation (12, 24, 36, 48, 72 h; data shown for only 48 h), the cells were observed under confocal microscope using Hybrid detector (HyD) at 561 nm to check the fluorescence from RFP protein. Similar experiments were conducted for pORF2-VLPs encapsidated with 5′-methyl-G-HEV(*1*–*249* *nt*)-*HBsAg* RNA and Indirect immunofluorescence assay was performed and [7] observed under inverted fluorescent microscope (Nikon TE2000-U) for the expression of HBsAg protein. The entry and internalization of pORF2 f-VLP’s (produced from ORF2-linker EGFP protein) has been previously demonstrated by us in Huh-7 cells [1]. However, in the present experiments, due to the lower concentration (250 nM) of RNA-VLP complex, immunofluorescent staining with anti-pORF2 could not be observed in RNA-VLP complex infected cells.

**Figure 3:**
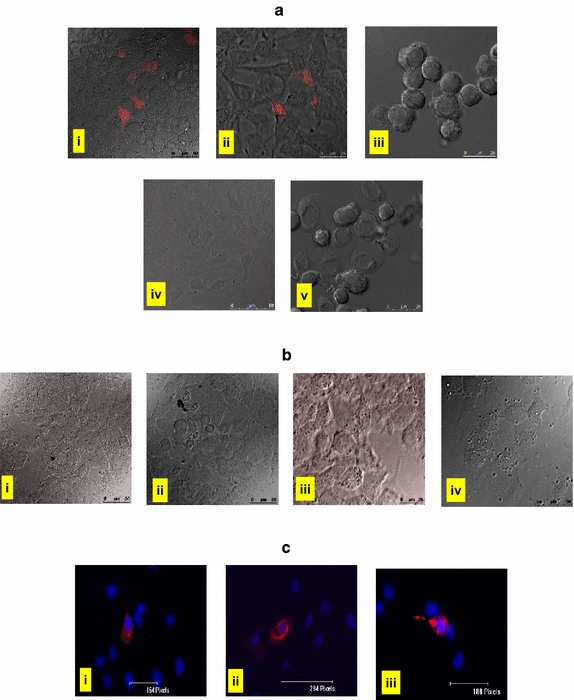
Internalisation of HEV-VLPs in different cell lines. **a** Confocal microscopy images for checking the expression of RFP in RNA-VLP [5′-methyl-G-HEV(*1*–*249* *nt*)-*RFP RNA* and *pORF2 VLP’s*] incubated *i* Huh7, *ii* A549, *iii* HeLa, *iv* Vero and *v* SiHa cells. **b** Confocal microscopy images for RFP fluorescence in Huh7 cells incubated with *i* 5′-methyl-G-HEV (*1*–*249* *nt*)-*RFP* RNA only; RNA-VLP complexes prepared from *ii* 5′-methyl-G-HEV (*199*–*444* *nt*)-*RFP* RNA and pORF2-VLPs, *iii* 5′-methyl-G-HEV (*1*–*249* *nt*)-*RFP* RNA and Δ112aa pORF2-VLPs, *iv* 5′-methyl-G-HEV(*1*–*249* *nt*)-*RFP RNA*, annealed with complementary oligo (HEV, 150–200 nt) and pORF2-VLPs. **c** Fluorescent microscopy images (*i*–*iii*) of Immunofluorescence assay for HBV surface antigen (HBsAg) in Huh 7 cells, incubated with HEV(1–249 nt)-*HBsAg*-RNA-pORF2 VLPs. Indirect immunofluorescence assay was performed for detecting HBsAg expression using anti-HBsAg primary antibody followed by Alexa-546 conjugate secondary antibody.

It was observed that RNA-VLP complex [prepared from 5′-methyl-G-HEV(*1*–*249* *nt*)-*RFP RNA* and *pORF2 VLP’s*] specifically entered liver cells (Huh7; 34.25%) and lung cells (A549; 31.88%) as shown by red fluorescence of expressed RFP (48 h post incubation) as well as expression of HBsAg protein, visualized by indirect immunofluorescence with anti-HBsAg antibody (48 h post incubation, Figure 3ci–iii). However, no signal was observed when Vero, SiHa and HeLa cells were used, indicating a cell specific entry of HEV VLP’s (Figure 3ai–v).

Parallel control experiments were performed, where Huh7 cells were incubated with only *5′*-*methyl*-*G*-*HEV* (*1*–*249* *nt*)-*RFP* RNA (Figure 3bi), and RNA-VLP complexes prepared from *5′*-*methyl*-*G*-*HEV* (*199*–*444* *nt*)-*RFP* RNA and pORF2-VLPs (Figure 3bii), *5′*-*methyl*-*G*-*HEV* (*199*–*444* *nt*)-*HBsAg* RNA and pORF2-VLPs and *5′*-*methyl*-*G*-*HEV* (*1*–*249* *nt*)-*RFP* RNA and N-terminal truncated Δ1-112aa pORF2-VLPs (Figure 3biii). However, in either case, no signal for RFP/HBsAg could be observed till 72 h which (1) ruled out the possibility of any non-specific RNA entry and (2) showed the significance of N-terminal HEV encapsidation (1–249 ntd) signal in mediating VLP entry (Figure 3).

Another control experiment was performed where 5′-methyl-G-HEV (*1*–*249* *nt*)-*RFP* RNA was annealed with complementary oligo (HEV, 150–200 ntd) and then packaged with pORF2-VLPs, which again showed no RFP signal in Huh7 cells (Figure 3biv) indicating the critical requirement of secondary structure and single stranded RNA for binding to HEV VLPs.

### Balb/c mice immunization with HBsAg RNA-pORF2 VLP complex and generation of Humoral immune response

**Figure 4:**
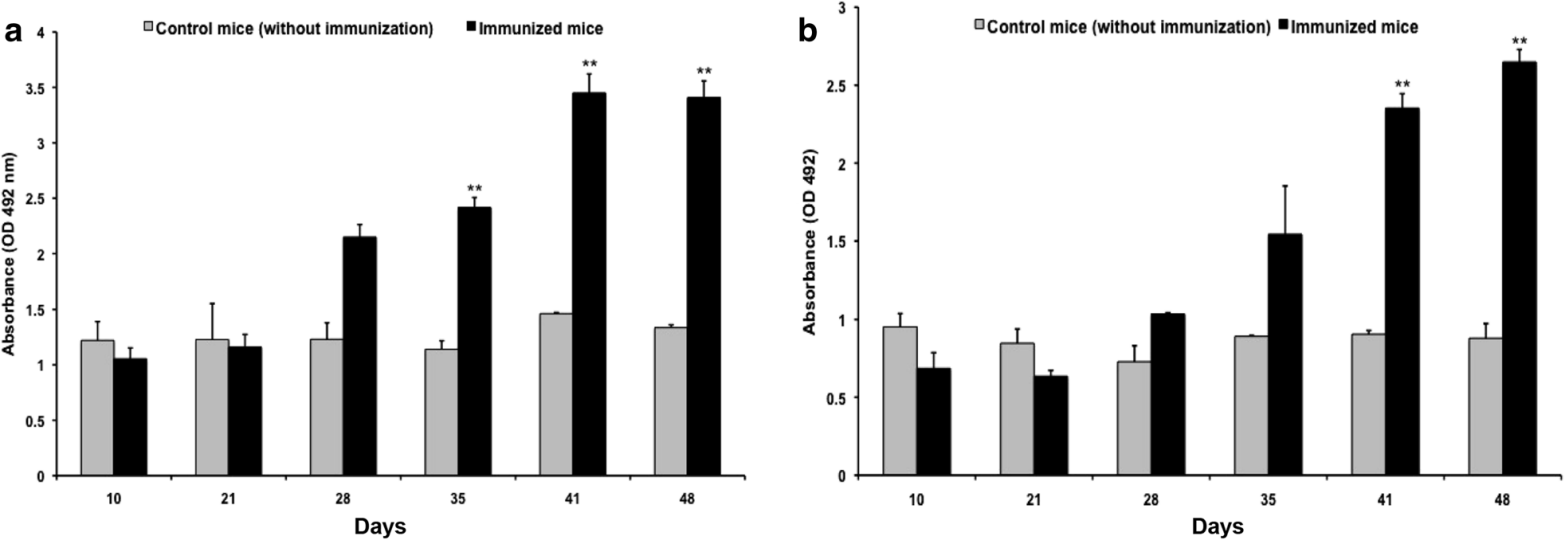
**a** Humoral response (against HBsAg antigen) raised in 5′-methyl-G-HEV-(1–249 nt)-*HBsAg*-pORF2 VLP complex immunized mice. The anti-HBsAg antibodies (quantitated by ELISA) were observed in *Balb/c* mice immunized with 5′-methyl-G-HEV (1–249 nt)-HBsAg RNA-pORF2 VLP comlpex. *Asterisks* show significant increase (**p < 0.01) in antibody titre (41st day post-immunization) as compared to un-immunized control mice. All the statistical analyses were done by multiple t test. **b** Humoral response (against HEV pORF2) raised in 5′-methyl-G-HEV-(*1*–*249* *nt*)-*HBsAg*-pORF2 VLP complex immunized mice. The anti-pORF2 antibodies (quantitated by ELISA) were observed in *Balb/c* mice immunized with 5′-methyl-G-HEV (1–249 nt)-HBsAg RNA-pORF2 VLP complex. *Asterisks* show significant increase (**p < 0.01) in antibody titre (48th day post-immunization) as compared to un-immunized control mice. All the statistical analyses were done by multiple t test.

After VLP-mediated internalization of foreign RNA and its subsequent expression in the cultured cells, we further studied the expression and immune response elicited in mice following the administration of RNA-VLP complex. Four 6-week-old *Balb*/c mice were immunized with intramuscular injections (*in tibialis anterior*) of 100 µg pORF2-VLP encapsidated with *5′*-*metly*-*G*-*HEV* (*1*–*249* *nt*)-*HBsAg* RNA on days 0, 14, 25, 32, 39 and 45 and blood samples were collected on days 10, 21, 28, 35, 41, 48 and 52. Sera were screened by ELISA, as previously described [8], for the presence of antibodies, both towards HBV surface antigen protein and HEV pORF2. Blood samples from four unimmunized mice served as negative controls in ELISA. Antibody against HBsAg as well as HEV capsid protein were detected in the immunized mice. The experimental mice showed maximal titer of anti-HBsAg and anti-pORF2 antibody on 41 and 48 days, respectively. (Figure 4a, b). Significant increase (**p < 0.01, *t* test) in antibody titre was observed for both anti-pORF2 and anti-HBsAg antibodies in immunised mice, as compared to un-immunized control mice.

Humoral immune response could be raised against both HEV pORF2 (monomeric unit of VLPs) and HBsAg protein (expressed from delivered hetrologous RNA), indicating the acceptance and processing of VLP-RNA complex as an effective antigen by host immune system. To our knowledge, this is the first direct demonstration of use of synthetic HEV VLPs for delivering RNA coding for a protein that acts as a potential immunogen in vivo.

## Abbreviations

HEV: Hepatitis E virus
VLP: virus like particles
HBsAg: Hepatitis B surface antigen
RFP: red fluorescent protein
ELISA: enzyme linked immunosorbent assay
ORF: open reading frame
nt: nucleotide
